# Draft Genome Sequence of Two Marine Plantactinospora spp. from the Gulf of California

**DOI:** 10.1128/genomeA.00436-18

**Published:** 2018-05-24

**Authors:** Luis Contreras-Castro, Luis A. Maldonado, Erika T. Quintana, Luciana Raggi, Alejandro Sánchez-Flores

**Affiliations:** a Laboratorio de Bioprospección en Actinobacterias, Escuela Nacional de Ciencias Biológicas (ENCB), Instituto Politécnico Nacional (IPN), Mexico City, Mexico; b Facultad de Química, Universidad Nacional Autónoma de México (UNAM), Mexico City, Mexico; c Instituto de Investigaciones Agropecuarias y Forestales, Universidad Michoacana San Nicolás de Hidalgo, Morelia, Mexico; d Unidad Universitaria de Secuenciación Masiva y Bioinformática (UUSMB), Instituto de Biotecnología, UNAM, Cuernavaca, Mexico

## Abstract

Plantactinospora sp. strains BB1 and BC1 were isolated in 2009 from sediment samples of the Gulf of California from among almost 300 actinobacteria. Genome mining of their ∼8.5-Mb sequences showed the bioprospecting potential of these rare actinomycetes, providing an insight to their ecological and biotechnological importance.

## GENOME ANNOUNCEMENT

The genus Plantactinospora within the class Actinobacteria currently contains seven validly described species (1–7), all of them recovered either in association with plants in China or from swamp forests in Thailand. None of them have been related to the marine environment yet. Plantactinospora spp. are Gram stain-positive and aerobic bacteria, which form extensive substrate mycelia that bear single or conglomerate spores, with little or no aerial mycelia. The genus has been identified mainly by its morphological and chemotaxonomical properties due to its high level of 16S rRNA similarities between its closely related members and to the genera Micromonospora, Salinispora, and Polymorphospora (>98.6%) (8).

Although the biotechnological potential of plantactinosporae has not yet been fully evaluated, the way in which they are related to members of the genus Salinispora could provide a first clue to its metabolic capabilities. Since its description in 2005 as the first marine obligate actinobacterial genus, Salinispora has proven to be a very promising source of secondary metabolites (9, 10).

Strains BB1 and BC1 were part of a previous study reporting the isolation of almost 300 actinobacteria (11) from the Gulf of California. Preliminary analyses have suggested that the isolates belonged to the family Micromonosporaceae. A phylogenetic reconstruction based on 16S rRNA gene sequences clustered the two strains within the genus Plantactinospora. EzTaxon (12) identified both BB1 and BC1 as being closely related to P. veratri, a species recovered from the root of Veratrum nigrum (5), with 99.17% and 98.97% 16S rRNA gene similarities, respectively.

The genomes of BB1 and BC1 were sequenced by ChunLab (Seoul, South Korea) using the Illumina MiSeq sequencing platform. The obtained reads for BB1 and BC1 were assembled in single chromosomes with SPAdes version 3.1.1 (13). Both genomes share a size of nearly 8.5 Mb. GC content values were also found to be extremely similar at 72.37% and 72.38%, respectively. Two-way average nucleotide identity values (14) indicated a 98.73% similarity, suggesting that both isolates belong to the same species.

The genomes were annotated with a pipeline running GeneMark under heuristic parameters (15) and an adapted Trinotate annotation workflow (16). The numbers of predicted open reading frames were 7,322 and 7,271, with 64 tRNAs and 65 tRNAs for BB1 and BC1, respectively, and 6 rRNAs for both. Mining of the two genomes using antiSMASH version 3.0 (17) found 83 and 84 potential secondary metabolite-related clusters for BB1 and BC1, respectively. The antiSMASH suite predicted the presence of gene clusters related to the production of erythrochelin, fortimicin, friulimicin, gentamicin, jagaricin, landepoxcin, lymphostin, naphtomycin, pradimicin, meilingmycin, taromycin, thiolutin, viomycin, and yatakemycin for the genome of BB1 and compound K-252a, erythrochelin, fortimicin, friulimicin, landepoxcin, lymphostin, meridamycin, naphtomycin, orfamide, pradimicin, sisomicin, taromycin, thiolutin, viomycin and yatakemycin for the genome of strain BC1, among others predicted by the Web tool NapDos (18).

### Accession number(s).

This whole-genome shotgun project has been deposited in GenBank under the accession no. CP028158 and CP028159. The versions described in this paper are the first versions, CP028158.1 and CP028159.1.
